# 3D Contrast Enhancement-MR Angiography for Imaging of Unruptured Cerebral Aneurysms: A Hospital-Based Prevalence Study

**DOI:** 10.1371/journal.pone.0114157

**Published:** 2014-12-02

**Authors:** Jing Li, Bixia Shen, Chao Ma, Li Liu, Li Ren, Yibin Fang, Dongwei Dai, Shiyue Chen, Jianping Lu

**Affiliations:** 1 Department of Radiology, Changhai Hospital of Shanghai, the Second Military Medical University, Shanghai, 200433, China; 2 Department of neurosurgery, Changhai Hospital of Shanghai, the Second Military Medical University, Shanghai, 200433, China; Fraunhofer Institute for Cell Therapy and Immunology, Germany

## Abstract

**Background and Purpose:**

Contrast enhanced MRA (CE-MRA) can help to overcome the limitations of other techniques to clearly display the details of cerebral aneurysms at 1.5-T MR system. We investigated the prevalence of unruptured cerebral aneurysms (UCAs) using three dimensional (3D) CE-MRA in a tertiary comprehensive hospital in China.

**Materials and Methods:**

The cases were prospectively recorded at our hospital between February 2009 and October 2010. 3D CE-MRA, interpreted by 2 observers blinded to the participants’ information, was used to identify the location and size of UCAs and to estimate the overall, age-specific, and sex-specific prevalence.

**Results:**

Of the 3993 patients (men: women = 2159∶1834), 408 UCAs were found in 350 patients (men: women = 151∶199). The prevalence was 8.8% overall (95% CI, 8.0–10.0%), with 7.0% for men (CI, 6.0–8.0%) and 10.9% for women (CI, 9.0–12.0%). The overall prevalence of UCAs was higher in women than in men (*P<*0.001) and increased with age both in men and women. Prevalence peaked at age group 75–80 years. Forty-two patients (11.7%) had multiple aneurysms, including 10 (2.9%) male patients and 32 (9.1%) female patients. The most common site of aneurysm was the carotid siphon, and most lesions (71.3%) had a maximum diameter of 3−5 mm.

**Conclusion:**

This hospital-based prevalence study suggested a high prevalence (8.8%) of UCAs and most lesions (71.3%) had a maximum diameter of 3–5 mm observed by 3D CE-MRA. Because the rupture of small cerebral aneurysms was not uncommon, an appropriate follow-up care strategy must be formulated.

## Introduction

The presence of an intracranial aneurysm is a serious medical condition that would lead to permanent neurological deficit or death if the aneurysm ruptures. The 30-day mortality rate might reach 45%−75% due to aneurysmal subarachnoid hemorrhage (SAH) [1]–[5]. As such, cerebral aneurysms remain an intractable public health issue. However, most patients (91%) have no obvious positive signs, or only some non-specific symptoms, until the aneurysm ruptures [6]. Based on different imaging techniques, a series of studies reported a widely varying prevalence of unruptured cerebral aneurysms (UCAs) ranging from 0.1%−8.4% [7]–[19], which may reflect methodological discrepancies among the studies, the non-specific presentations of UCAs or the differences in age- and sex- distributions of the sample population.

Several factors have been found to be associated with an increased risk of rupture, including aneurysmal features (size, location and shape), demographic characteristics and medical histories (smoking, drinking, hypertension and family history of SAH) [12], [20]. The key way to radically reduce the high mortality and morbidity caused by the rupture of intracranial aneurysms is to detect UCAs’ anatomic features using effective imaging techniques, and then prophylactically implement intervention therapy. Digital subtraction angiography (DSA) is generally considered the gold standard for the diagnosis of intracranial arterial disease. However, invasiveness, time consumption and some small but significant neurological complications are still existed in DSA examination, which makes it less than ideal as a screening tool and for the extended follow-up of patients with UCAs [21], [22]. Multidetector computed tomography (MDCT) and the 3D algorithms for image post-processing provided high spacial resolution, time saving and suitability for patients with some MR contraindications (those with pacemakers and intravascular stents) [23]–[25]. However, disadvantages including the use of a nephrotoxic contrast agent, allergic reaction and ionizing radiation [26]–[28] limited the application of MDCT in the follow-up of patients who need multiple inspections. In terms of 3D time-of-flight (TOF) -MRA, the advantage of this technique is that a contrast agent is not required. However, imaging time for TOF MRA is long resulting in limited anatomic coverage and high susceptibility to motion artifacts at 1.5-T MR system. In addition, blood flow conditions (especially complex flow and slow flow) and thrombus in UCAs are relevant to the heterogeneity of blood signal. These would cause both an underestimation of the size of aneurysm and an inaccurate depiction of the UCA’s morphology [29]–[32]. Most of these problems can be overcome with CE-MRA, which is not relying on blood flow to create intravascular signal. A contrast agent is introduced to shorten the T1 relaxation of blood, thus the intra-luminal signal is not dependent on flow conditions [33]. The aim of the study was to evaluate the detection rate of UCAs by using three-dimensional (3D) CE-MRA in a tertiary comprehensive hospital.

## Materials and Methods

### Study design and process

This prospective study was approved by our Institutional Review Board, Shanghai Changhai Hospital Ethics Committee. Signed written informed consent was obtained from all participants before the examinations. 4022 consecutive cases were recruited and underwent head and neck 3D CE-MRA between February 2009 and October 2010 at a single academic institution. Patients were eligible for enrollment if they met the following criteria: (1) patients visited our hospital for neurological conditions, such as headache, dizziness and motor weakness; (2) signed the informed consent; (3) aged 20–80 years; (4) cooperated with the collection of their past medical histories and blood biochemical indices. Exclusion criteria were: (1) refusal to participate in the study; (2) pacemaker implantation and metallic implants; (3) allergic history of Gd-DTPA; (4) coma, epileptic seizure and obnubilation; (5) acute ischemic stroke; (6) patients with chronic kidney disease (stage 4 or 5); (7) pregnancy and (8) claustrophobia. After analyzing the participants’ basic information, we found that most of the patients came from south of the Yangtze River in China, whose age (15−59, 67.0%;>60, 33.0%) and sex ratios (male, 54.1%; female, 45.9%) closely matched those in the sixth national census (15−59, 70.14%;>60, 13.26%; male, 51.27%; female, 48.73%) of China.

The demographic characteristics, past and family medical history and lifestyle risk factors of the participants were recorded. Research contents included history of SAH, blepharoptosis, hypertension, hyperlipidemia, diabetes, smoking (defined as having smoked ≤10 cigarettes/day and smoked > 10 cigarettes/day) and drinking (defined as consumption of 1–40 g/day and > 40 g/day) [34].

### Imaging Technique

MRI examinations were performed on a 1.5-T imaging system (Avanto, Siemens, Erlangen, Germany). CE-MRA was performed in the coronal plane using a 3D fast low-angle-shot sequence with TR = 3.0 ms; TE = 1.03 ms; FOV = 228 mm; flip angle = 25°; 88 slices of 1 mm thickness; matrix, 256 × 256. Contrast bolus timing was determined directly by fluoroscopy. CE-MRA was performed after intravenous injection of 0.2 mmol/kg bodyweight Gd-DTPA. Contrast administration was injected through a 20-gauge cannula at a rate of 3 ml/s into an antecubital vein, using an automatic double-syringe injector. All contrast agent injections were followed by a 15−20 ml saline flush injected at the same rate. Patients were asked to hyperventilate before scanning and to hold their breath on inspiration during image acquisition. No significant complications occurred during or after MRA in any patients in the study.

### Image Post-processing

The acquired image data sets were then transferred to a workstation (Avanto, Siemens, Erlangen, Germany), in which reconstruction with maximum intensity projection (MIP) and volume rendering (VR) was done. A single artery was made visible by manually cutting and then highlighting the relevant segment from multiple angles on VR images. The quality of images was assessed by two radiologists in consensus using a 3-point scale in which:1 = lesion scarcely visible (artifacts/noise impairs the diagnosis); 2 = good depiction of an aneurysm; 3 = excellent depiction of an aneurysm. Only images with a quality score of 2 and 3 were used for assessment. Seventeen participants were excluded for insufficient image quality.

### Image Review

**Figure 1:**
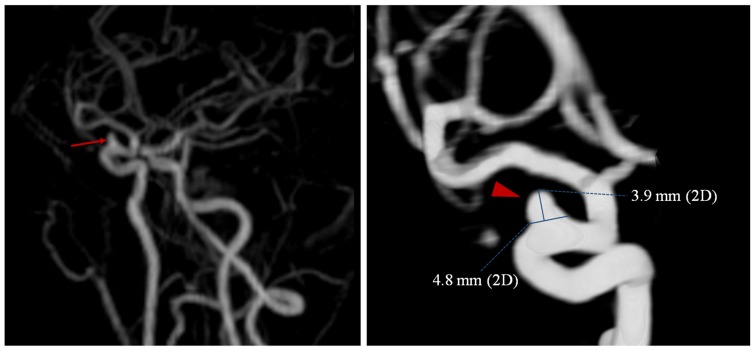
A 3-dimensional contrast enhancement magnetic resonance angiography image reveals a small aneurysm in the right siphon carotid artery on the MIP image (arrow), which is more clearly delineated on the VR image (4.8 mm × 3.9 mm).

All aneurysms that were 2 mm or more in largest dimension were included in the study. According to the rupture risk of aneurysms, UCA size was assessed utilizing five size categories (2−3 mm, 3−5 mm, 5−7 mm, 7−12 mm and ≥12 mm) [6], [35]. Two observers, blinded to the participants’ information, independently measured the size of aneurysms on volume rendered images (Figure 1). VR images allowed the appropriate threshold for window width and level to be set for the differentiation of small aneurysms with infundibula. The location was intuitively evaluated on MIP or VR images. Aneurysm sites were classified as internal carotid artery (including the posterior communicating artery), middle cerebral artery (including the M1-2 bifurcation), anterior cerebral artery (including the anterior communicating artery) and vertebrobasilar artery.

### Statistical Analysis

To calculate the appropriate sample size, a preliminary study (n = 400) was conducted that showed an initial prevalence of 10.5% (calculation formula: n = t^2^pq/d^2^, p = 10.5%, q = 1-p, d = 0.1p, t = 1.96). Accordingly, we calculated a sample size of 3410. Quantitative variables were reported as means ± standard deviation (SD) and qualitative variables as number and percentage. The *t*-test was used to compare continuous variables. The chi-square was used to compare categorical variables. All *P-*values were two-sided and were considered statistically significant if they were 0.05 or less. In addition, the Cochran-Armitage trend test, conducted by using SAS (version 9.1.3, SAS Institute, Cary, North Carolina) was adopted to examine whether a trend existed between age and the prevalence of UCAs. According to the measurement results of aneurysmal size by the two radiologists, the interobserver agreement was evaluated by the kappa statistics, which was performed by using SPSS, version 17.0 (SPSS Inc., Chicago, IL, USA).

## Results

### Patients

Among the 4022 cases that underwent head and neck 3D CE-MRA, 12 patients meeting the exclusion criteria were excluded from the study, and 17 patients’ images were rejected due to a lack of the required quality. The imaging data set therefore consisted of a total of 3993 consecutive patients (men: women = 2159∶1834; mean age, 53.8 years; age range, 20–80 years) who underwent MRA for various causes including a routine health check-up at outpatient’s clinic for further analysis. The clinical characteristics of the participants were summarized in Table 1.

**Table 1:** Clinical characteristics.

Characteristic	Aneurysm (n = 350)	No aneurysm (n = 3643)	Total (n = 3993)	P value
Mean age (SD), y	58.9(12.1)	54.4(12.6)	54.8(12.6)	<0.001
Female/male, n/n	199/151	1635/2008	1834/2159	<0.001
Smoking, n (%)	100(28.6)	1091(29.9)	1191(29.8)	0.71
≤10 cigarettes/day	45(12.9)	526(14.4)	571(14.3)	
>10 cigarettes/day	55(15.7)	565(15.5)	620(15.5)	
Alcohol use, n (%)	90(25.7)	1050(28.8)	1140(28.5)	0.44
1–40 g/day	67(19.1)	773(21.2)	840(21.0)	
>40 g/day	23(6.6)	277(7.6)	300(7.5)	
Hypertension, n (%)	172(49.0)	1740(47.8)	1912(47.9)	0.66
Diabetes, n (%)	35(10.5)	389(10.9)	424(10.9)	0.80
Hyperlipidemia, n (%)	94(28.5)	953(27.1)	1047(27.2)	0.59
History of SAH, n (%)	7(2.0)	20(0.5)	27(0.7)	0.002
Blepharoptosis, n (%)	5(1.4)	36(1.0)	41(1.0)	0.44

### Prevalence of UCAs

The interobserver agreement for the size of aneurysm was good, with a kappa statistic of 0.818. Of the 3993 patients (men: women = 2159∶1834), 408 UCAs were found in 350 patients. The prevalence was 8.8% overall (95% CI, 8–10%), with 7.0% for men (CI, 6–8%) and 10.9% for women (CI, 9–12%). The overall prevalence of UCAs was higher in women than in men (*P<*0.001) and increased with age in both men and women (peaked at ages 75–80). A total of 42 patients (11.7%) had multiple aneurysms, with 10 (2.9%) male patients and 32 (9.1%) female patients (Table 2).

**Table 2:** Age- and sex-specific prevalence of unruptured cerebral aneurysms.

Age (years)	Men			Women				Total	
	Total	Aneurysms		Total	Aneurysms		Total	Aneurysms	
		Participants (n)	Proportion (95% CI)		Participants (n)	Proportion (95% CI)		Participants (n)	Proportion (95% CI)
20–25	34	0	/	41	2	4.9(0–0.12)	75	2	2.7(0–0.06)
26–35	135	5	3.7(0–0.07)	112	5	4.5(0.01–0.08)	247	10	4.0(0.02–0.07)
36–45	402	20	5.0(0.03–0.07)	299	22	7.4(0.04–0.10)	701	42	6.0(0.04–0.08)
46–55	603	40	6.6(0.05–0.09)	509	43	8.4(0.06–0.11)	1112	83	7.5(0.06–0.09)
56–65	593	40	6.7(0.05–0.09)	527	70	13.3(0.10–0.16)	1120	110	9.8(0.08–0.12)
66–75	323	36	11.1(0.08–0.15)	294	46	15.6(0.11–0.20)	617	82	13.3(0.11–0.16)
75–80	69	10	14.5(0.06–0.23)	52	11	21.2(0.11–0.33)	121	21	17.4(0.11–0.24)
Total	2159	151	7.0(0.06–0.08)	1834	199	10.9(0.09–0.12)	3993	350	8.8(0.08–0.10)
*P-value of trend*			<0.0001			<0.0001			<0.0001

### Aneurysmal Characteristics

The distribution of aneurysm locations and sizes for the 3993 patients was summarized in Table 3. The most common site of aneurysm was carotid siphon where 340 aneurysms (83.3%) occurred. Most lesions (71.3%) had a maximum diameter 3−5 mm, followed by 5−7 mm (57, 14.0%), 2−3 mm (33, 8.1%), 7−12 mm (23, 5.6%), and ≥12 mm (4, 1.0%). The mean maximum diameter of the aneurysm was 4.4±1.9 mm (Table 4).

**Table 3:** Location of Unruptured Cerebral Aneurysms. Numbers in parentheses are percentages.

Location	Men	Women	Sub-total	Total
ICA				
C1	6	9	15 (3.7)	
SCA	128	212	340(83.3)	
C5+EIC	17	5	22(5.4)	377(92.4)
MCA	2	7	9(2.2)	
ACA	5	4	9(2.2)	
VA-BA	4	9	13(3.2)	
Total	162	246	408	

ICA, internal carotid artery; SCA, siphon carotid artery; EIC, extracranial internal carotid; MCA, middle cerebral artery; ACA, anterior cerebral artery; VA, vertebral artery; BA, basilar artery.

**Table 4:** Proportion of different sizes of aneurysms.

Size	Men	Women	Total
<3 mm	18(11.1)	15(6.1)	33(8.1)
3–5 mm	111(68.5)	180(73.2)	291(71.3)
5–7 mm	27(16.7)	30(12.2)	57(14.0)
7–12 mm	5(3.1)	18(7.3)	23(5.6)
≥12 mm	1(0.6)	3(1.2)	4(1.0)
total	162	246	408
x–(s), mm	4.5(2.3)	4.4(1.6)	4.4(1.9)

## Discussion

Before rupture, a large number of patients with UCAs are asymptomatic or only taking on some mild non-specific symptoms, including headache (36%), followed by ischemic cerebrovascular events (17.6%), and cranial nerve deficits (15.4%) [11]. Except for acute headache and isolated cranial nerve III palsy, which are seen as warning signs, others are considered as non-specific symptoms [36]. Without the help of vascular imaging’s noninvasive techniques, some inexperienced doctors may miss or erroneously diagnose these symptoms as unknown (24%), primary migraine syndromes (21%), meningitis and systemic infections (when associated with fever), stroke, and hypertensive crises [37]. This is why a fraction of patients with UCAs miss early detection and ultimately leading to underestimation of the true prevalence of UCAs.

In the study, we found the hospital-based prevalence of UCAs was 8.8% using 3D CE-MRA, which was higher than the findings of many previous studies [7]–[17]. In a recent study using 3D TOF-MRA (3-T), Ming-Hua Li et al. reported the overall prevalence of UCAs was 7.0% in Chinese adults aged 35–75 years (mean age, 53.1 years) [18], which might reveal the aneurysmal prevalence of the general population. Roughly consistent with our results, Igase et al. reported the rate of 8.4% acquired by 3D TOF-MRA on 3-T MRI in patients with neurological conditions in Japan [19]. There were some plausible explanations for the high detection rate of UCAs. First, the study was based on the patients with mild non-specific neurological symptoms in one hospital, which might produce a higher incidence of UCAs than the general population. Second, for large UCAs and UCAs with small necks, TOF-MRA may underestimate detection rate of UCAs and inaccurate measurement of UCAs’ size [30], [32], [33]. Third, we used the single-artery highlighting approach to detect UCAs [18], which was conducive to show aneurysms from multiple angles. Fourth, for 1.5-T MR system, some researchers proved that 3D CE-MRA was superior to 3D TOF-MRA in the visualization of cerebral aneurysm [30], [39]. Although 3-T TOF MRA provided greater image quality and an accordingly high accuracy and sensitivity of more than 95% for small cerebral aneurysms [38], 3-T 3D CE-MRA is superior to 3D TOF-MRA for assessment of sac shape, detection of aneurysmal neck and visualization of branches originating from the sac or neck itself if the size of the aneurysm is greater than 13 mm [32]. The variety of blood flow regimes ranging from laminar to complex and/or turbulent as well as thrombosis in various phases of development within the larger aneurysms may produce very heterogeneous signal intensity resulting in potential errors in image interpretation.

The cumulative rate of rupture of cerebral aneurysm less than 10 mm without a history of aneurysm rupture had been estimated as 0.05% per year [11]. Recently, investigators of the International Study of Unruptured Intracranial Aneurysms reported aneurysms of 7 mm or larger were associated with a significantly increased risk of rupture [6]. Villablanca JP et al. found that growth of asymptomatic UCAs of all sizes was not uncommon and was associated with a higher rupture risk and concluded imaging follow-up of all patients with aneurysms including those whose aneurysms are smaller than the current 7 mm treatment threshold [40]. In the current study, it was noting that the proportion of UCAs with a diameter of 3−5 mm is 71.3%, which is much higher than the other previous reports [18], [19]. The high detection rate is a valuable warning indicator for all the patients with neurological conditions to receive follow-up care.

The result of the present study found the most common location of UCAs was the internal carotid artery, which was consistent with previous reports [7], [11], [17]–[19]. These results might partly result from the exclusion of ruptured aneurysms, which were frequently located in the anterior or posterior communicating arteries [6], [7]. Furthermore, the internal carotid artery was susceptible to the influences of skull base and calcification of vascular wall [41], [42] which might be the reason that CTA underestimated the incidence of UCAs located in the internal carotid artery in some studies [6], [13]. In addition, VR imaging combined with the single-artery highlighting approach and views from arbitrary angles may improve the detection rate of UCAs located in carotid siphon, because vessels lying near the sphenoid sinus were subject to susceptibility artifact and the detectability of aneurysms was greatly reduced by the presence of overlapping vessels [43].

Previous studies found that smoking, hypertension, excessive alcohol consumption, history SAH, female sex (especially postmenopausal women) and age were the major risk factors for the rupture of UCAs [12], [20]. However, in the current study, except age, sex and history SAH, the association between hypertension, smoking, alcohol consumption and the prevalence of UCAs were not statistically significant. The exclusion standard of patients with ruptured aneurysms might cause the different findings in the current study. Therefore, in order to identify the relationship between those aforementioned risk factors and the prevalence of UCAs, design of some specifically longitudinal studies were needed.

Some researchers have found an association between the use of certain gadolinium-based contrast agents in patients with severe renal impairment and nephrogenic systemic fibrosis [44]. Therefore, we set an exclusion standard for those patients with chronic kidney disease in the design of the study. Additionally, contrast agent of CE-MRA has both fewer allergies and lower dose than that of CE-CTA examination [45].

Some limitations of our study should be noted. First, without validation using DSA, when there was high suspicion of an aneurysm being present, a high estimate of accuracy could result due to observer expectation bias. Second, the proportion of participants over 60 years of age (33%) was higher than that of the general population (13.26%). Crucially, much of the literature considered that the incidence of UCAs increase with age in men and women [6]–[7], [14], [18], [35]. It can be speculated that this partly explains the relatively high incidence in our study.

In conclusion, this hospital-based prevalence study suggested a high prevalence (8.8%) of UCAs and most lesions (71.3%) had a maximum diameter of 3−5 mm observed by 3D CE-MRA. Because the rupture of small cerebral aneurysms was not uncommon, an appropriate follow-up care strategy must be formulated.
